# The role of MLKL in Hepatic Ischemia-Reperfusion Injury of Alcoholic Steatotic Livers

**DOI:** 10.7150/ijbs.67533

**Published:** 2022-01-01

**Authors:** Hao Chen, Tara McKeen, Xiaojuan Chao, Allen Chen, Fengyan Deng, Hartmut Jaeschke, Wen-Xing Ding, Hong-Min Ni

**Affiliations:** Department of Pharmacology, Toxicology and Therapeutics, University of Kansas Medical Center, Kansas City, Kansas 66160, USA.

**Keywords:** Alcoholic liver disease, Cell death, Inflammation, Ischemia-reperfusion injury, Liver injury, Necroptosis.

## Abstract

Alcohol-related liver disease (ALD) and non-alcoholic fatty liver disease (NAFLD) are the primary causes of chronic liver disease in western countries. Liver transplantation is currently one of the most efficient approaches to save patients with liver failure, which is often associated with hepatic ischemia-reperfusion (IR) injury. IR injury is exacerbated by hepatic steatosis, yet the mechanism remains elusive. Necroptosis is a form of regulated cell death mediated by receptor-interacting protein kinase 1 (RIP1), RIP3 and mixed lineage kinase domain-like (MLKL) protein, which has been implicated in the pathogenesis of ALD and NAFLD. Though necroptosis plays an important role in IR injury of high fat diet - induced steatotic livers, the role of necroptosis in IR injury of ethanol - induced steototic livers has not been investigated. In the present study, we used chronic plus binge alcohol (Gao-binge) feeding followed by IR surgery to investigate IR liver injury with ethanol-associated steatosis. We found that the levels of key necroptotic proteins MLKL and RIP3 increased in alcohol-fed mouse livers. Moreover, we observed increased liver injury after IR in control diet-fed mice, which was further exacerbated by alcohol feeding based on serum alanine aminotransferase (ALT) levels and TUNEL staining of necrotic cells. Hepatic neutrophil infiltration also increased in alcohol-fed mice after IR surgery. However, deletion of *Mlkl* did not protect against IR liver injury in alcohol-fed mice compared with matched wild-type mice. In conclusion, alcoholic steatosis promotes IR injury, which seems to be independent of MLKL-mediated necroptosis.

## Introduction

Alcohol-related liver disease (ALD) and non-alcoholic fatty liver disease (NAFLD) are the two major forms of chronic liver disease having become worldwide burdens 1. The prevalence of ALD is estimated to be about 8.1% of adult population; the number of patients with advanced ALD and the number of individuals on the waiting list for liver transplantation both having increased dramatically in the US since 2001 2. The pathogenesis of ALD includes simple steatosis followed by fibrosis with potential for progression to more severe conditions including alcoholic hepatitis, cirrhosis, and hepatocellular carcinoma 3, 4.

Hepatic ischemia-reperfusion (IR) injury is an important cause of liver injury clinically, which is associated with many clinical situations such as liver transplantation, liver resection surgeries, and systemic shock 5. The main mechanisms contributing to hepatic IR injury include depletion of oxygen and adenosine triphosphate (ATP), increased hepatic inflammation, mitochondrial uncoupling, and resultant oxidative stress after reperfusion 6. Liver transplantation, which remains the most effective treatment option for end-stage liver disease (ESLD) including as a result of advanced ALD, is, however, limited by inadequate organ supply 7. Marginal liver grafts, including steatotic livers, have sometimes been accepted for liver transplantation because of the organ shortage 8. It has been demonstrated that the donor livers with moderate-to-severe steatosis (approximately 30%-60% macrosteatosis) have a high risk of IR injury and increased mortality or other complications following liver transplantation 8, 9. However, how fatty liver increases IR injury remains unclear, which has halted the use of fatty livers in liver transplantation.

Accumulating evidence suggests that necroptosis is critical in the pathogenesis of inflammatory liver diseases including NAFLD, ALD, and hepatic IR injury 10-15. Necroptosis is a form of regulated cell death, which is mediated by three key molecules: receptor-interacting protein kinase 1 (RIP1), receptor-interacting protein kinase 3 (RIP3), and mixed lineage kinase domain-like protein (MLKL) 16, 17. Activation of necroptosis shares a common pathway with apoptosis through death receptor activation 18-20. In the absence of functional caspase-8 or FADD, RIP1 and RIP3 form an amyloid necrosome and become activated, leading to phosphorylation and activation of MLKL, the downstream executioner of necroptosis. The activated phosphorylated MLKL translocates to plasma membrane where they form oligomers or polymers, which leads to the perforation of the cell membrane and subsequent lytic cell death 20-23. Pores formed by MLKL result in release of damage-associated molecular patterns (DAMPs) and other inflammatory factors including IL-6 and IL-1β and illustrating the important role of necroptosis in NAFLD, ALD, hepatic IR injury, and other inflammatory diseases 8, 24. As such, genetic deletion of RIP3 protects against steatosis and liver injury in both ALD and NAFLD 12, while inhibition of necroptosis through genetic deletion of MLKL or pharmacological means ameliorates steatosis and liver injury in NAFLD 25, 26. Our previous studies 27 demonstrated that hepatic steatosis caused by western diet (WD) exacerbates IR injury. However, deletion of MLKL and RIP3 protects IR injury in WD - induced steatotic mouse livers. Very limited publications on hepatic IR injury of alcoholic fatty liver exist, and these studies only focused mainly on hepatic inflammation 28-30. The role of necroptosis especially MLKL, as a terminal executioner of necroptosis, in hepatic IR injury of alcoholic fatty liver has not been studied 31.

Here, we used a murine model of alcohol-induced hepatic steatosis and characterized its effects on hepatic IR injury. We found that chronic plus binge ethanol feeding increased hepatic RIP3 and MLKL as well as hepatic steatosis, which exacerbated IR injury with increased neutrophil infiltration. However, *Mlkl^-/-^* mice failed to protect from IR injury in livers with alcohol-induced steatosis. These findings may suggest that MLKL may be dispensable in IR injury of alcoholic fatty livers.

## Materials and Methods

### Reagents

Antibodies used in this study were MLKL (#SAB1302339) and β-actin (#A5441) from Sigma-Aldrich, Sodium Potassium ATPase (#ab76020) from Abcam, RIP3 (#2283) from Prosci, MPO (#PP023AA) from Biocare Medical, Horseradish peroxidase-conjugated secondary antibodies were purchased from Jackson ImmunoResearch Laboratory. Lieber-DiCarli ethanol diet (Bio-Serv #F1258SP) and control diet (Bio-Serv #F1259SP) were purchased from Bio-Serv.

### Animals

C57BL/6N *Mlkl^-/-^* mice were generated using CRISPR/Cas-mediated genome engineering method by Cyagen (CA) as described previously 27. *Mlkl^+/-^* mice were crossed with each other to obtain *Mlkl^-/-^*and matched *Mlkl^+/+^* (wild type, WT) mice. Two to 3-month-old male mice were used in this study. All mice (maximum 5 mice per cage) were maintained with a 12-hour light/dark cycle of humane care. All procedures have been approved by the Institutional Animal Care and Use Committee of the University of Kansas Medical Center.

### Mouse hepatic ischemia-reperfusion model

We used the chronic plus binge alcohol feeding (“Gao-binge”) murine model to perform the experiments 32. In brief, male mice of 2-3 months-old were fed 5% Lieber-DeCarli ethanol liquid diet or pair-fed control diet (CD) for 10 days, and on the last day of feeding, mice were gavaged with 4.5g/kg ethanol or maltose control for 8 hours followed by hepatic ischemia and reperfusion surgery 33. Hepatic ischemia was established by occluding the portal triad with clamp (hepatic artery, portal vein, and bile duct) of the left lobe, which provides approximately 70% of the total body's blood supply to the liver, for 45 minutes. The clamp was removed and followed by reperfusion for 6 or 24 hours. Sham control mice were performed the same procedures without vessel occlusion. Mice were euthanized following reperfusion and blood samples and liver tissues were collected. Liver injury was determined by measuring serum alanine aminotransferase (ALT) activities.

### Histology and immunohistochemistry analyses

Mouse liver tissues were fixed with 10% formalin and embedded in paraffin followed by histopathological analysis. Paraffin-embedded liver sections were stained with hematoxylin and eosin (H&E) for pathological evaluation. Immunohistochemistry staining for myeloperoxidase (MPO), a neutrophil marker, was performed 34. The number of cells with MPO positive staining was quantified by counting twenty different fields under microscopy using 200× magnification. Terminal deoxynucleotidyl transferase dUTP nick end labeling (TUNEL) staining was performed to evaluate cell death as described previously 12. Cryo-liver sections were used for Oil Red O staining of hepatic lipids as we previously described 35.

### Plasma membrane extraction and Western Blotting analysis

Total liver protein lysates were extracted using radioimmunoprecipitation assay buffer (RIPA buffer containing 1% NP40, 0.1% sodium dodecyl (lauryl) sulfate, 0.5% sodium deoxycholate in phosphate-buffered saline) supplemented with protease inhibitor cocktail (Bimake). Plasma membrane fraction of liver tissues were extracted by using a Minute^TM^ plasma membrane and cell fractionation kit according to the manufacturer's instruction (Invent Biotechnologies, SM-005). Protein (30 μg) was separated on a SDS-PAGE gel and transferred to a PVDF membrane. Membranes were incubated with appropriate primary and secondary antibodies. SuperSignal plus chemiluminescent substrate was then used to visualize the results (ThermoFisher). Densitometry analysis was performed with the Un-Scan-It software and normalized to either β-actin or GAPDH for total protein or Sodium Potassium ATPase for plasma membrane protein. All the densitometry data were presented as mean ± SEM.

### Quantitative real time polymerase chain reaction (qRT-PCR) analysis

Total RNA was extracted from mouse livers using Trizol^®^ reagent (Thermo Fisher). cDNA was synthesized by Maxima H minus reverse transcriptase (Thermo Fisher). qRT-PCR was performed on a BioRad CFX384 real-time PCR system (Bio-Rad) using SYBR Green PCR mix (Bimake). The expression levels of *Tnfa*, *Il1b*, *Mip1a*, *Mip1b*, *Mcp1* and *Mip2* were quantified and *Atcb* was used as an internal control. The fold change of mRNA was expressed as 2^-∆∆Ct^. The primers were listed in **Table 1**.

### Hepatic TG analysis

Hepatic TG in mice were extracted with chloroform/methanol solution. The TG contents were measured using TG liquid reagent according to the manufacturer's instruction (Pointe Scientific).

### Statistical analysis

All experimental data were expressed as means ± standard error of the mean (SEM) and subjected to Student's t-test or one-way ANOVA with Holm-Sidak post hoc test where appropriate. *p<0.05 was considered significant.

## Results

### Increased key necroptosis proteins in ethanol-induced steatotic mouse livers

**Figure 1:**
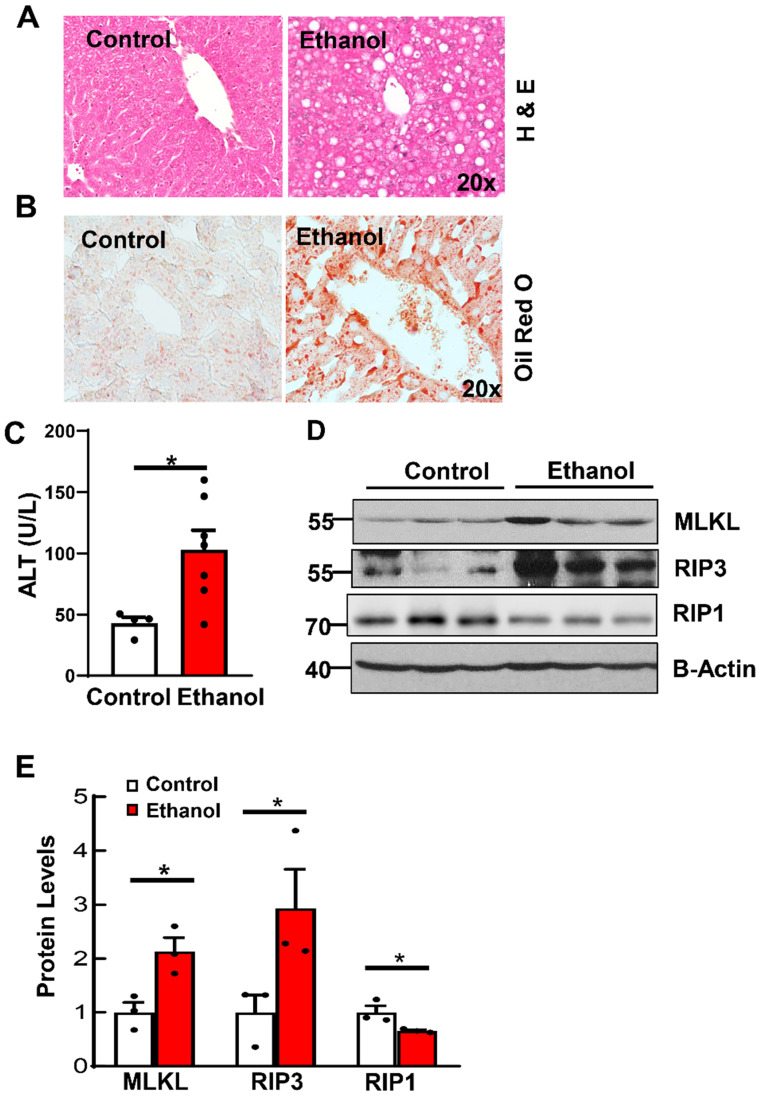
** Ethanol induces steatosis and increases necroptosis proteins in mouse livers.** Wildtype mice were pair-fed with CD or ethanol for 10 days followed by binge for another 8 hours. Liver steatosis was assessed by hematoxylin and eosin (H & E) staining (A) and Oil Red O staining (B). (C) Serum alanine aminotransferase (ALT) activities were measured in mice fed with ethanol. (D) Total proteins were extracted from mouse liver homogenates. Western blotting for RIP1, RIP3 and MLKL was analyzed. (E) Densitometry analysis was performed. Values were expressed as mean ± SEM (n=3 per group). *p < 0.05 vs CD group. CD, control diet.

To investigate the role of necroptosis in alcohol-induced fatty liver, we first established a chronic plus binge alcohol mouse model. As shown in **Figures 1A and 1B**, results from H&E and Oil Red O staining showed increased steatosis in ethanol-fed mouse livers. Levels of serum ALT were significantly increased in ethanol-fed mice compared to CD-fed mice (**Figure 1C**). Relatedly, western blot analysis revealed increased levels of hepatic RIP3 and MLKL in ethanol-fed mice compared to CD-fed mice (**Figures 1D and 1E**). Unlike RIP3 and MLKL, the hepatic RIP1 level decreased in ethanol-fed mouse livers compared to CD-fed mouse livers (**Figure 1D and 1E**). These data indicate that ethanol feeding increases couple key necroptosis proteins and steatosis in mouse livers.

### Ethanol feeding and Ischemia-reperfusion (IR) increase MLKL plasma membrane translocation in mouse livers

**Figure 2:**
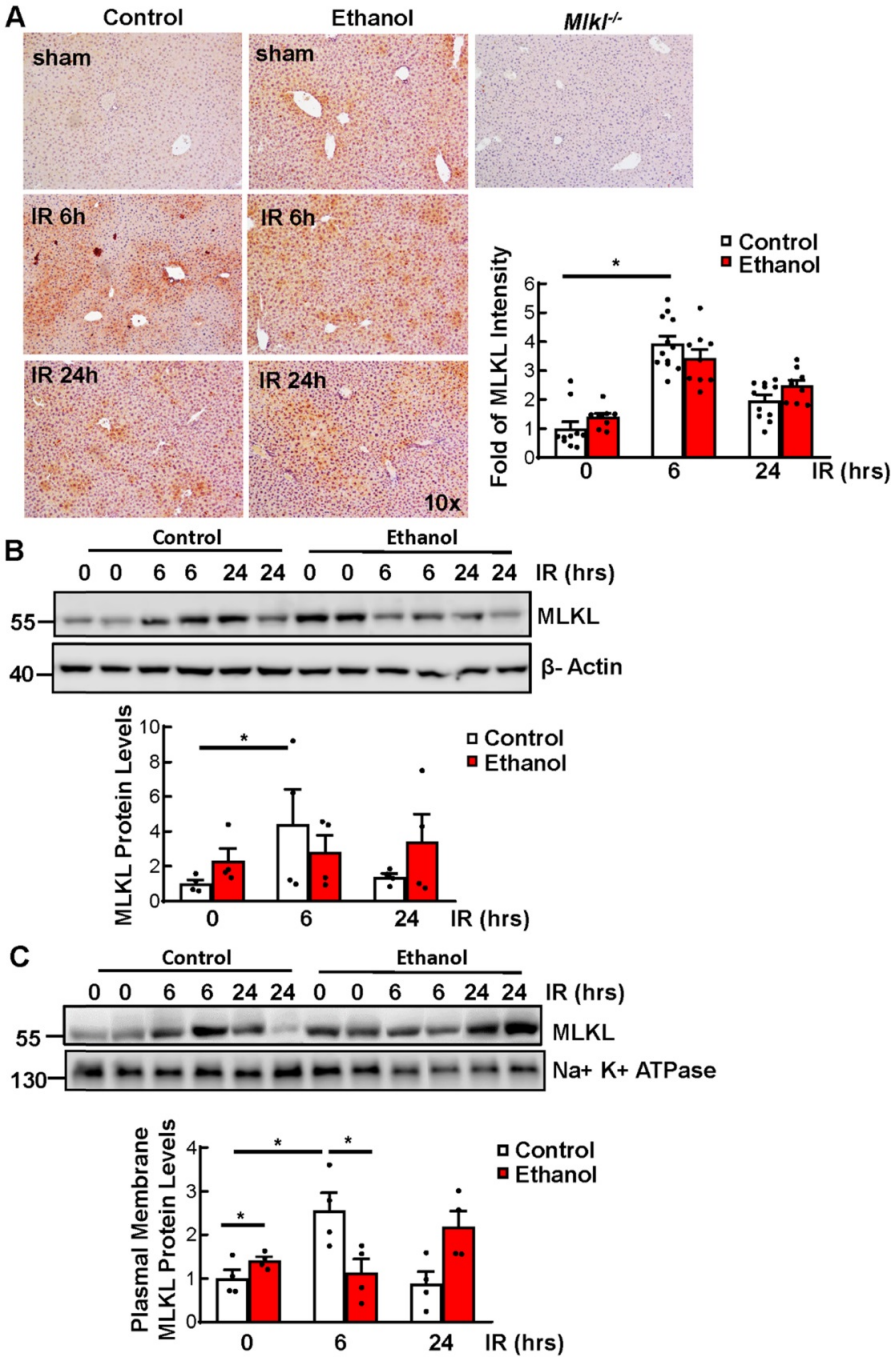
**Ethanol and Ischemia-reperfusion (IR) increased MLKL plasma membrane translocation in mouse livers.** Wildtype mice were fed with CD or chronic plus binge ethanol and were subjected to 45 minutes of ischemia and 6 and 24 hours of reperfusion. (A) The protein level of MLKL was detected by immunohistochemistry staining in livers and quantified. Three mice per group and three to five fields per mouse were quantified. (B) Total proteins were extracted from mouse livers and the protein levels were analyzed by immunoblots. Densitometry analysis was performed. (C) Plasma membranes were extracted and immunoblot analysis was performed and quantified. Values were expressed as mean ± SEM (n=4 per group). *P < 0.05.

We have previously shown that IR increases MLKL plasma membrane translocation 27. We found that IR increased the level of MLKL in CD-fed mice. Ethanol alone also increased MLKL in livers of sham mice compared to those of CD-fed sham mice, though the levels of MLKL did not further increase under IR as shown by immunohistochemistry staining and immunoblot analysis (**Figures 2A and 2B**). Since MLKL-plasma membrane translocation is a critical step for triggering necroptosis, we next determined the MLKL levels on plasma membrane. We found that IR increased MLKL plasma membrane translocation in CD-fed mice. Ethanol alone slightly increased MLKL plasma membrane translocation, though this was not further increased by IR (**Figure 2C**). These results indicate that IR or ethanol feeding alone increases MLKL plasma membrane in mouse livers, but the levels of plasma membrane MLKL are not further elevated in IR of ethanol-fed mouse livers which could probably be due to the non-specific degradation which is caused by the extensive injury.

### Ethanol feeding exacerbates ischemia and reperfusion liver injury in mice independent of MLKL

**Figure 3:**
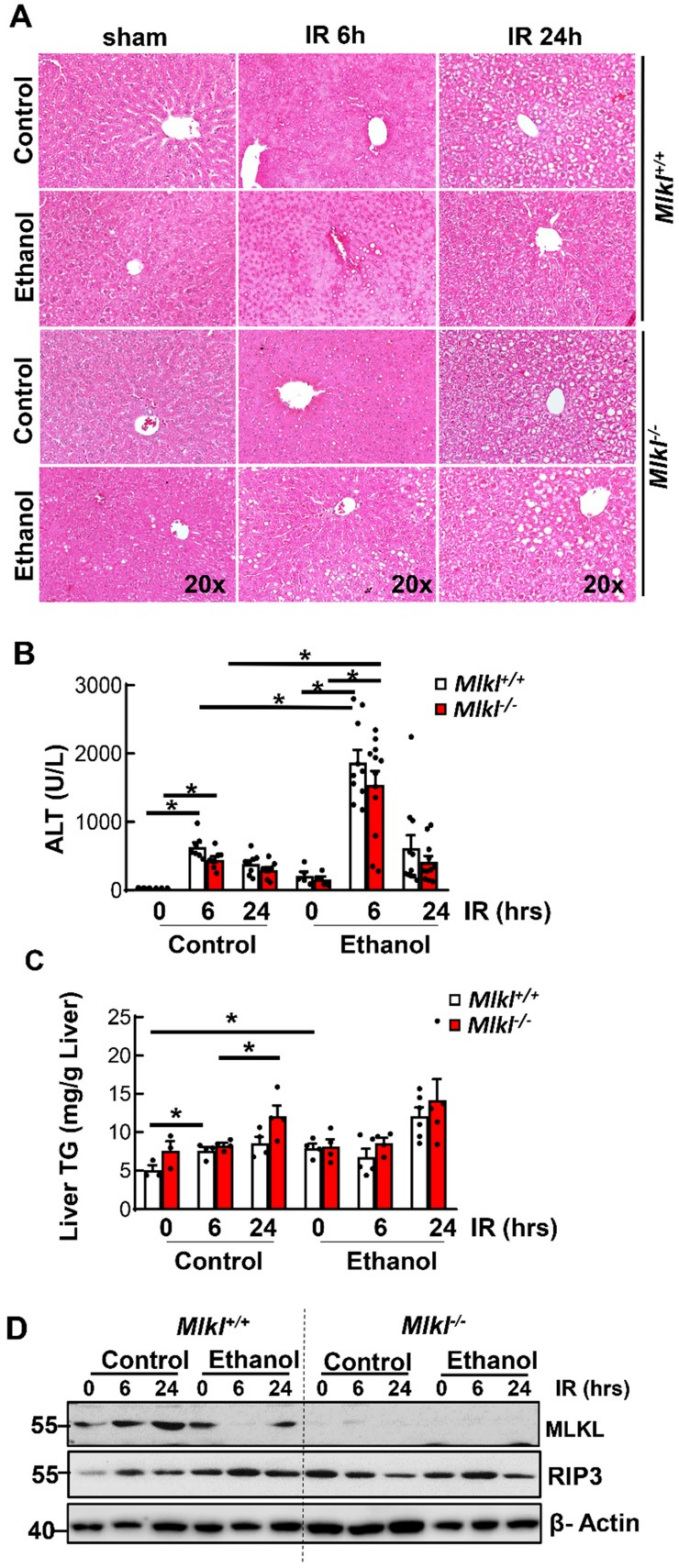
**Hepatic IR injury in *Mlkl^+/+^* and *Mlkl^-/-^* mice fed with CD or WD.** *Mlkl^-/-^* and matched *Mlkl^+/+^* mice were fed with CD or ethanol and were subjected to IR surgeries as described in Figure 2 legend. (A) Liver injury was assessed by H&E staining. (B) Serum alanine aminotransferase (ALT) activities were analyzed. Values were expressed as mean ± SEM (n=3-10 per group). *P < 0.05. (C) Hepatic TG were extracted from mouse livers and quantified. Values were expressed as mean ± SEM (n=3-4 per group). *P < 0.05. (D) Total proteins were extracted from mouse liver homogenates. Equal amount of protein from different samples (n=3-10 per group) were pooled and subjected to immunoblot analysis.

**Figure 4:**
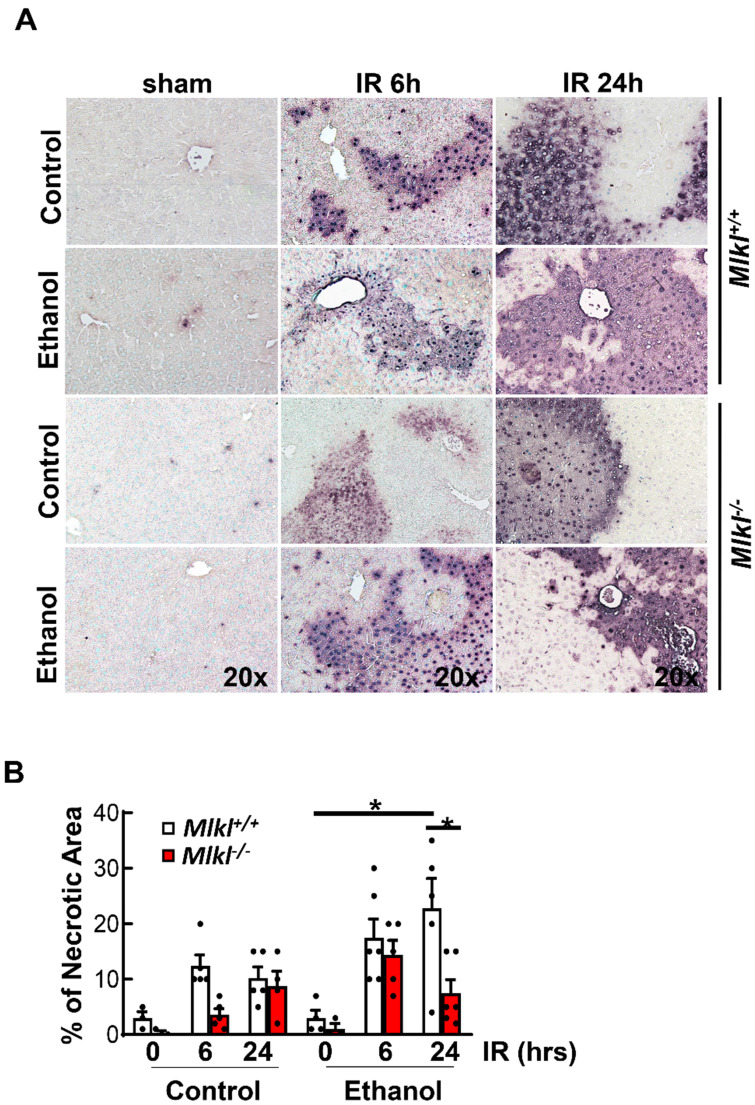
**Increased TUNEL staining in *Mlkl^+/+^* and *Mlkl^-/-^* mice fed with CD or WD with IR.** *Mlkl^-/-^* and matched *Mlkl^+/+^* mice were fed with CD or ethanol and were subjected to IR surgeries as described in Figure 2 legend. (A) Liver injury was assessed by TUNEL staining. (B) The area of necrosis with TUNEL positive staining was measured. Values were expressed as mean ± SEM (n=3-10 per group). *P < 0.05.

Results from H&E staining showed that IR mainly caused hepatocyte necrosis and ethanol-fed mice had larger necrotic areas compared to CD-fed mice in the liver (**Figure 3A**). Serum ALT activities were significantly higher in ethanol-fed mice than CD-fed mice after IR. However, levels of serum ALT were not significantly lower in *Mlkl^-/-^* mice than in *Mlkl^+/+^* mice after IR in either CD or ethanol-fed mice (**Figure 3B**), which was consistent with H & E staining. Ethanol and IR increased hepatic TG in mice, but IR didn't further increase hepatic TG (**Figure 3C**). Furthermore, there was no difference of hepatic TG content between *Mlkl^+/+^* and *Mlkl^-/-^* mice (**Figure 3C**). Similarly, while IR and ethanol increased protein levels of RIP3 and MLKL, IR didn't further increase necroptosis proteins even decreased in ethanol-fed mouse livers at 6 hours post perfusion (**Figure 3D**). Deletion of MLKL were confirmed by immunoblot (**Figure 3D**). TUNEL staining showed diffuse patterns after IR, indicating necrotic cell death but not apoptosis similar to acetaminophen-induced necrosis 36 (**Figure 4A**). Since the TUNEL positive areas were coincided with the necrotic areas in H&E staining, we quantified areas of necrosis using TUNEL staining. The TUNEL positive areas were not significantly different between *Mlkl^-/-^* and *Mlkl^+/+^* mice after IR regardless of CD or ethanol feeding (**Figure 4B**). These data indicate that ethanol exacerbated hepatic IR injury and MLKL-mediated necroptosis may be dispensable in the pathogenesis of IR and ethanol-induced liver injury.

### Decreased hepatic neutrophil infiltration and inflammation in *Mlkl^-/-^* mice after IR

**Figure 5:**
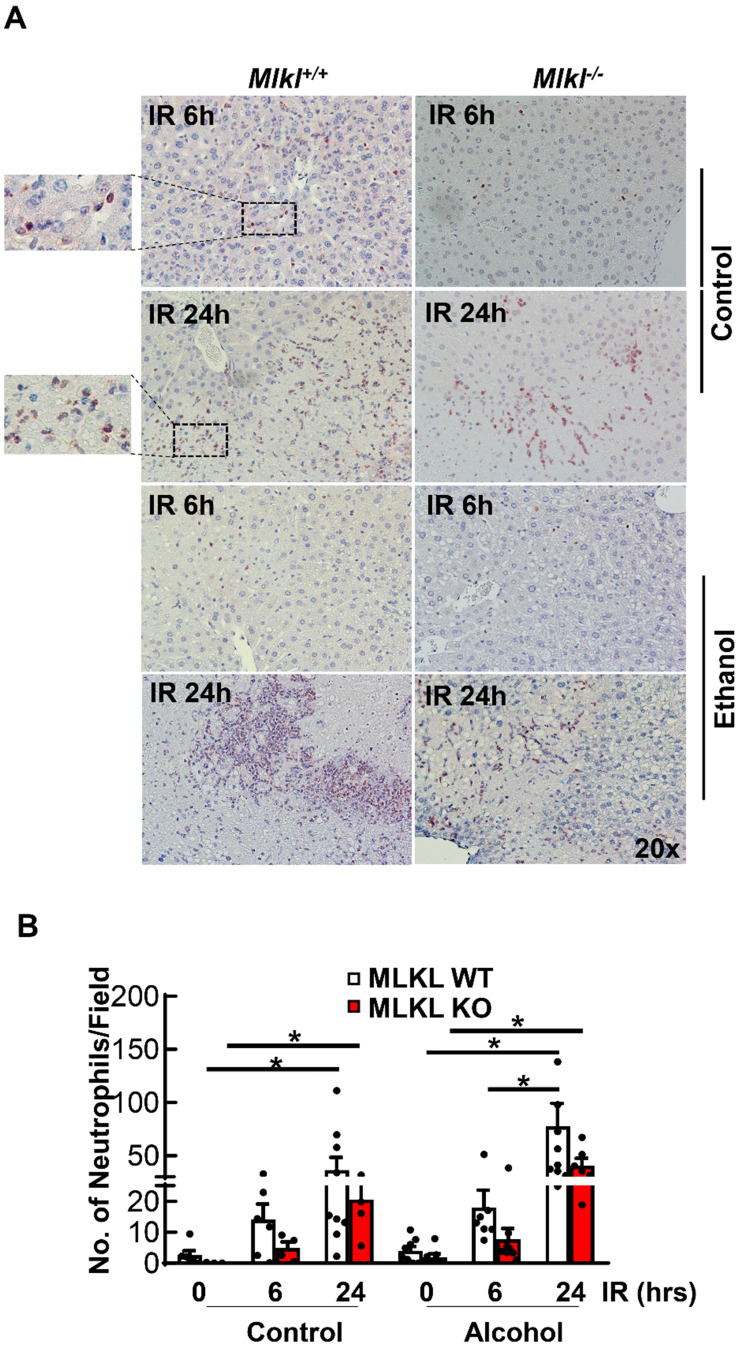
**Detection of neutrophil infiltration in *Mlkl^+/+^* and *Mlkl^-/-^* mouse livers following IR surgery.** *Mlkl^-/-^* and matched *Mlkl^+/+^* mice were fed with CD or ethanol and were subjected to IR surgeries as described in Figure 2 legend. (A) Neutrophils were stained with Myeloperoxidase (MPO) on liver sections after 6 and 24 hours of reperfusion. (B) MPO positive cells were counted in 20 fields per liver. Values were expressed as mean ± SEM (n=3-9 per group). *P < 0.05.

**Figure 6:**
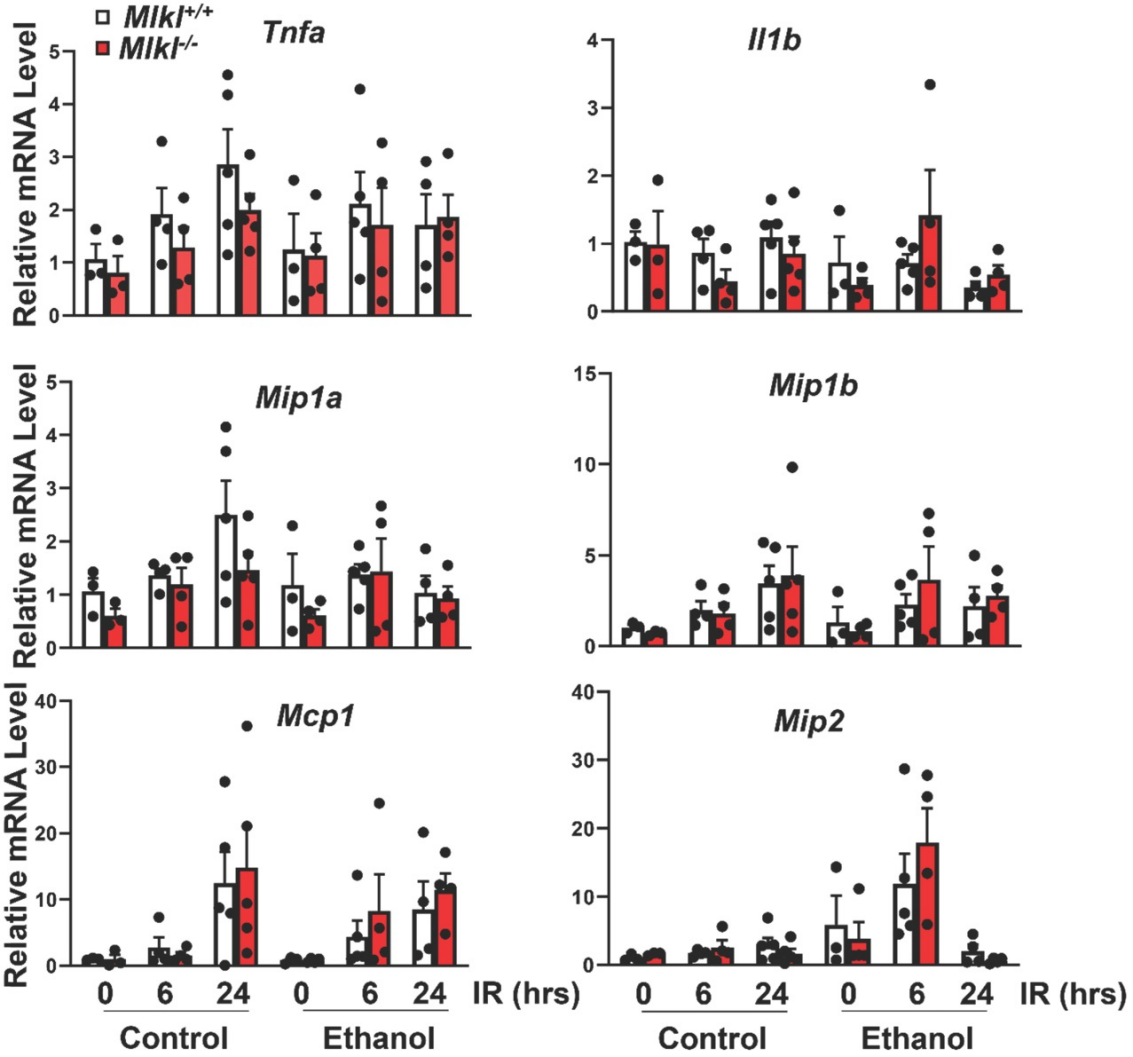
** The mRNA expression levels of pro-inflammatory genes in *Mlkl^+/+^* and *Mlkl^-/-^* mouse livers following IR surgery.** *Mlkl^-/-^* and matched *Mlkl^+/+^* mice were fed with CD or ethanol and were subjected to IR surgeries as described in Figure 2 legend. The mRNA levels of several pro-inflammatory genes were quantified by RT-PCR. *Actb* was used as an internal control. Values were expressed as mean ± SEM (n=3-5 per group). *Il1b*, Interleukin 1 beta; *Mcp1*, monocyte chemotactic protein 1; *Mip1a*, macrophage inflammatory protein 1 alpha; *Mip1b*, macrophage inflammatory protein 1 beta;* Mip2,* macrophagy inflammatory protein 2; *Tnfa*, tumor necrosis factor alpha.

Neutrophil activation is an important cause of hepatic IR injury 37. MPO staining is widely used to stain neutrophils in mouse livers. Few MPO-positive neutrophils were present in sinusoids in both CD and ethanol-fed mice of sham groups (**Figures 5A**). The number of neutrophils extravasated into the hepatic parenchyma increased at 6 hours after reperfusion, which were further increased at 24 hours in both *Mlkl^+/+^* and *Mlkl^-/-^* mice. The number of neutrophils tended to be lower but was not statistically different in *Mlkl^-/-^* mice compared to *Mlkl^+/+^* mice. Ethanol feeding did not change the number of infiltrated neutrophils in either *Mlkl^+/+^* or *Mlkl^-/-^* mice (**Figure 5B**). The expression levels of pro-inflammatory genes were also quantified by qRT-PCR (**Figure 6**). IR increased mRNA levels *Tnfa* as early as 6 hours post perfusion.IR also increased mRNA levels of chemokine genes *Mip1a*, *Mip1b*, *Mcp1* and *Mip2*. *Mip2* further increased in ethanol feeding groups. However, there were no differences between *Mlkl* WT and KO mouse livers. These data further suggest that MLKL may not be involved in neutrophil infiltration and inflammation in IR injury of either CD or ethanol-fed mice.

## Discussion

Hepatic IR injury is a common clinical complication. Studies from rodent models have consistently shown that hepatic steatosis by either NAFLD or ALD exacerbates hepatic IR injury 28, 29, 37-40. In NAFLD-associated IR injury studies, either genetic models of obesity such as *ob/ob* mice or high fat diet-induced steatosis models have been utilized 37-39. However, studies to examine IR injury in ALD are quite limited and most of these studies employed a chronic ethanol feeding rodent model 28, 29, 40. In the present study, we used a chronic plus binge ethanol model followed by IR surgeries to induce hepatic steatosis and measure IR injury severity in mice. Consistent with other studies 28, 29, 40, our data showed that ethanol feeding induced hepatic steatosis and exacerbated IR injury.

Our group and others have previously demonstrated that necrosis but not apoptosis is the predominant type of cell death in hepatic IR 27, 33, 41. Necroptosis, a well-studied form of regulated cell death, is initially identified as a shift of cell death away from apoptosis towards necrosis under conditions when TNF-α binds to its receptor TNFR1 while apoptosis is inhibited by caspase inhibitors. When caspases, in particular caspase-8, are inhibited, RIP1 and RIP3 bind to each other to form an amyloid-like structure, which then phosphorylates MLKL. Next, phosphorylated MLKL translocates to the plasma membrane, increasing plasma membrane permeability and resulting in induction of necroptosis 16, 42. RIP3-MLKL-mediated necroptosis is involved in various liver diseases including NAFLD, ALD, and hepatic IR injury. Afonso et al.^26^ found that inhibition of necroptosis pharmacologically by Necrostatin-1, a RIP1 inhibitor, or by genetic deletion of RIP3, protected against high-fat choline-deficient (HFCD) diet- or methionine choline-deficient (MCD) diet-induced NAFLD. However, others reported that deletion of RIP3 exacerbated high-fat diet-induced NAFLD but had no effects on NAFLD induced by a high fat, fructose, and cholesterol (FFC) diet 10, 25. These different results are likely due to the use of different diets and feeding duration to induce NALFD, as differing lipid/fatty acid composition may trigger different cell death signaling pathways. Intriguingly, a recent study showed that deletion of MLKL protected against FFC diet-induced liver injury through correcting impaired autophagy induced by FFC 25. Palmitic acid (a saturated fatty acid), in a RIP3-independent pathway, promoted the translocation of MLKL to the autophagosome membrane and inhibited autophagy. These findings suggest that the key players of necroptosis may have non-necroptosis roles, which may complicate data interpretation.

In addition to playing critical roles in the pathogenesis of NAFLD, necroptosis has also been implicated in hepatic IR injury. Administration of Necrostatin-1 attenuated hepatic IR injury in mice 13. In addition, we recently demonstrated that *Mlkl^-/-^* protected against hepatic IR injury of steatotic livers in mice fed with the western diet (WD) 27. In ALD, inhibition of necroptosis by deletion of RIP3 protected against chronic plus binge alcohol-induced liver injury in mice 12. In the present study, we found that chronic plus binge ethanol feeding increased the protein levels of both RIP3 and MLKL, as well as plasma membrane translocation of MLKL in mouse livers. However, deletion of MLKL, the terminal executor of necroptosis, had only mild and non-significant protective effects against hepatic IR injury in ethanol-fed mouse livers. These findings are different from our previous findings that deletion of MLKL alleviated hepatic IR injury of steatotic livers in WD-fed mice 13. The difference could be caused by different steatotic models utilized in the studies, as well as the lipid composition and the extent of inflammation in NAFLD vs ALD as discussed above. Notably, a recent study by Miyata et al. found that MLKL played a differential role in ALD and NAFLD 14. Although MLKL protein level and plasma membrane translocation were both significantly increased in HFF-fed mouse livers, there was no change in MLKL protein level or plasma membrane translocation in chronic plus binge ethanol mouse livers. Liver injury as measured by ALT slightly decreased in *Mlkl^-/-^* mice fed with ethanol which was similar to our findings. Future lipidomic and metabolomic studies to directly compare NAFLD and ALD may yield interesting clues to help further understand the complex mechanisms involved in these diseases.

It should be noted that the contribution of excessive inflammatory response due to microcirculatory dysfunction to hepatic IR injury is well documented 43-46. Specifically, resulting hepatic neutrophil infiltration is thought to be important in hepatic IR injury, previous studies having shown that activation and infiltration of neutrophils after reperfusion generate reactive oxygen species (ROS) 37, 46. Our data also showed that the numbers of neutrophils are elevated in necrotic areas of the liver during the reperfusion period. In addition, we have found that the numbers of neutrophils are slightly increased in ethanol-fed mouse livers compared to CD-fed mouse livers, though this number did not further increase after IR in ethanol-fed mouse livers. These data suggest that other mechanisms rather than neutrophil infiltration contribute to IR injury exacerbation in ethanol-fed mice. Increased microcirculatory disturbances in NAFLD may contribute to IR injury 37. Besides its necroptotic function, MLKL may contribute to vascular function 47, 48. Deletion of MLKL has been shown to rescue vascular defects and lethality in *Casp8^-/-^* or *Fadd^-/-^* mice 47. MLKL may also regulate vascular adhesion molecule expression 48. It is possible that ethanol may cause mild vascular disturbances compared to WD-fed mice, which needs to be further investigated.

Pyroptosis, another form of regulated cell death, is one of the most important immune responses to pathogen infection or endogenous challenge 49, 50. The canonical pathway of pyroptosis initiates from an intracellular pattern recognition receptor (PRR) that detects a pathogen-associated molecular pattern (PAMP) or an endogenous danger signal (DAMP). Several intracellular PRRs, including NOD-like receptor (NLR) members (NLRP1, NLRP3, NAIP-NLRC4) and non-NLR receptors (AIM2 and PYRIN), can scaffold a canonical inflammasome complex that recruits and activates caspase-1 in an apoptosis-associated speck-like protein containing a CARD (ASC) adaptor-dependent or independent manner. Active caspase-1 cleaves Gasdermin D (GSDMD) and liberates GSDMD-N domain, which exhibits a potent pore-forming activity to cause membrane lysis. Active caspase-1 also cleaves pro-IL-1β and pro-IL-18 into their active forms, which are released from the membrane pore to cause inflammation 49, 50. Non-canonical pathway of pyroptosis initiates by LPS to active caspase-4, 5, and 11, which cleave GSDMD independent of inflammasome 49, 50. More recent studies also found that pyroptosis is activated through caspase-3-dependent pathway which activates GSDME 51. While excessive pyroptosis causes various inflammatory diseases like sepsis, pyroptosis also gains more attention in the pathogenesis of ALD and IR injury 52, 53. It is known that inflammation is associated with ALD and IR injury. Several pyroptosis proteins including NLRP3, ASC and caspase-1/4/11 were upregulated in patients with alcoholic hepatitis and in mice 54, 55. Inhibition of IL-1β or deletion of ASC and caspase-1 ameliorate inflammation, steatosis and liver injury in mice 56. Pyroptosis also increases in hepatic IR injury. Deletion of caspase 1 or caspase-1/11 attenuated IR injury in mice fed with high fat diet 57. Inhibition of inflammasome - induced pyroptosis with drugs also ameliorated hepatic IR injury in rats 58. However, the role of pyroptosis in hepatic IR injury in ALD is unknown and needs to be further investigated. In summary, chronic plus binge ethanol not only elevates levels of key necroptosis proteins RIP3 and MLKL as well as MLKL plasma membrane translocation, but also promotes hepatic IR injury. However, loss of MLKL does not protect against IR liver injury in ethanol-fed mice.

## Figures and Tables

**Table 1:** The lists of primers used in qRT-PCR.

Gene	Forward (5'-3')	Reverse (5'-3')
*Actb*	TGTTACCAACTGGGACGACA	GGGGTGTTGAAGGTCTCAAA
*Il1b*	GCCCATCCTCTGTGACTCAT	AGGCCACAGGTATTTTGTCG
*Mcp1*	CCAGCCTACTCATTGGGAT	GGGCCTGCTGTTCACAGTT
*Mip1a*	TGAGAGTCTTGGAGGCAGCGA	TGTGGCTACTTGGCAGCAAACA
*Mip1b*	AACACCATGAAGCTCTGCGT	AGAAACAGCAGGAAGTGGGA
*Mip2*	CTCAGAGGAAGACGATGAAG	GACGAGTTATCCCAGCCAAA
*Tnfa*	CGTCAGCCGATTTGCTATCT	CGGACTCCGCAAAGTCTAAG

## Abbreviations

ALD: Alcoholic Liver Disease
ALT: alanine aminotransferase
H&E: hematoxylin and eosin
IR: ischemia and reperfusion
KO: knockout
MLKL: mixed lineage kinase-like protein
MPO: Myeloperoxidase
NAFLD: Non-alcoholic Fatty Liver Disease
RIPA: radioimmunoprecipitation
RIP3: receptor-interacting protein kinase 3
TUNEL: Terminal deoxynucleotidyl transferase dUTP nick end labeling
TNF-α: tumor necrosis factor alpha
WT: wild-type
